# The Flip Side of the Coin: Giftedness in Pediatric Acute-Onset Neuropsychiatric Syndrome

**DOI:** 10.3390/children11121524

**Published:** 2024-12-16

**Authors:** Denise Calaprice, Ryan Terreri, Christopher Whitty, Ryan Whitty, Janice Tona

**Affiliations:** 1 Brain Inflammation Collaborative, Delafield, WI 53018, USA; 2 University of California, Los Angeles, CA 90095, USA; rcterreri@ucla.edu; 3 Thornton School of Music, University of Southern California, Los Angeles, CA 90007, USA; cwhitty@usc.edu; 4 Department of Computer Science, University of California, Berkeley, CA 94720, USA; rwhitty@berkeley.edu; 5 Department of Rehabilitation Science, University at Buffalo, Buffalo, NY 14214, USA; tona@buffalo.edu

**Keywords:** PANS, PANDAS, giftedness, memory, community-based survey

## Abstract

**Background/Objectives:** Individuals with Pediatric Acute-onset Neuropsychiatric Syndrome (PANS), an immune-modulated disorder, experience exacerbation-related neuropsychiatric symptoms, functional impairments, and high rates of developmental diagnosis. The literature describes links between giftedness and mental illness, and giftedness and autoimmune disorders. We sought to explore rates of giftedness among children with PANS as perceived by their caregivers, and to examine whether giftedness was related to PANS symptom severity, persistence, or duration. **Methods:** Data were extracted from a larger, 146-item survey, with 680 respondents meeting inclusion criteria of being a parent/guardian of a child with PANS and answering questions regarding perceived giftedness in empathy, social skills, verbal ability, reading, memory, math, creativity, or “other.” **Results:** In all, 604 respondents indicated some type of giftedness; the categories of giftedness were each endorsed by 30–57% of respondents. We found no significant associations between giftedness and severity of worst symptoms, persistence of symptoms, or length of time since symptom onset, once Bonferonni corrections were applied. Significantly more females than males were identified as gifted in creativity, but no other sex-related differences were seen. Thematic analysis of optional comments revealed three themes: (1) Elaboration on Types of Giftedness; (2) Objective Basis for Perceptions of Giftedness; and (3) Impact of PANS on Giftedness. **Conclusions:** The rate of giftedness reported by parents of PANS subjects in this study is much higher than would be expected in the general population, even when adjusting generously for potential overestimation. This study of the “flip side” of PANS should serve as impetus for future studies regarding giftedness in this population; a robust finding of exceptionally high rates of giftedness would have implications for diagnosis, interpretation of symptoms (for example, perfectionism and social challenges) and disease management.

## 1. Introduction

Pediatric Acute-Onset Neuropsychiatric Syndrome, or PANS, most often presents in childhood with neuropsychiatric symptoms including obsessions, compulsions, mood lability, restrictive eating, tics, sleep disturbance, and others, often with abrupt onset and frequently after infection [1,2,3]. Most PANS patients experience cognitive deterioration during exacerbations including brain fog/confusion, and problems with memory, concentration, executive function, and processing speed, along with speech deterioration [1,3,4,5]. The pathophysiology of PANS is widely recognized to be immunologic, with uncontrolled immune activation leading to basal ganglia inflammation observable via multiple brain imaging modalities (reviewed in [6]). Indeed, emerging evidence suggests that laboratory indicators of immune activation, systemic inflammatory symptoms, autoimmune/inflammatory diagnoses and family histories are so prevalent in this population that PANS might best be classified as a rheumatologic diagnosis with early and prominent neuropsychiatric manifestations [5,7,8].

Because of its pervasive symptomatology, PANS frequently arrests the ability of its victims to function at age-typical levels. High rates of developmental diagnosis, needs for school accommodation, and functional “lags” relative to peers have been reported in multiple cohorts of patients with sustained symptoms [1,9,10,11]. What the published data do not depict, however, is the perception by many clinicians and advocates that PANS patients, as a group, are not inherently cognitively deficient, but, to the contrary, frequently gifted but suffering from profound, though often reversible, disease-related debility (e.g., as related by D. Pohlman, Executive Director, PANDAS Network, pers. comm. 25 February 2024).

The perception of high rates of giftedness among PANS patients aligns with the known associations between neuropsychiatric disturbance, autoimmunity, and giftedness. The link between mental illness and giftedness has been recognized for millennia [12] and is supported by an abundance of formal evidence (reviewed in [13,14]). Less commonly appreciated, but also robustly evidenced, is the association between giftedness and autoimmunity [14,15,16,17,18,19]. For example, significantly increased rates of allergies and asthma have been reported among gifted children in numerous studies across several decades [20,21,22]. In one study of over 400 students whose Scholastic Aptitude Test (SAT) scores reflected reasoning abilities in the top 0.01%, Benbow found that approximately half reported allergies, asthma, and other immune disorders, significantly exceeding the expected prevalence [16,17]. More recently, an association between high IQ in children and an excessive inflammatory response to SARS-CoV-2 infection has been reported [23]. Clusters associating allergies, autoimmune disease, sensory sensitivity and high IQ have been found among individuals with ADHD [24,25], and among those with autism spectrum disorder (ASD) [26,27]. Despite the fact that PANS resides squarely at the intersection of psychiatric and autoimmune disease, however, a possible link between PANS and giftedness has never before been explored in a systematic fashion. An unexpectedly high rate of giftedness in this population would have significant implications for diagnosis, assessment, medical management, psychotherapeutic support, and occupational accommodations, and thus would be important to recognize.

Here, we present data regarding caregiver perceptions of the giftedness of PANS patients, and we explore correlations between perceived giftedness and disease history. Although criteria for the designation “gifted” vary, the National Association for Gifted Children considers “children who are in the top 10 percent in relation to a national and/or local norm” to be a good guide for identification and services [28]. The US federal definition of gifted children is “Students, children, or youth who give evidence of high achievement capability in areas such as intellectual, creative, artistic, or leadership capacity, or in specific academic fields, and who need services and activities not ordinarily provided by the school in order to fully develop those capabilities” [29]. Parents’ perceptions of giftedness (absent a specific definition), as studied here, are of course different from giftedness as assessed by schools, governments and/or objective standards. Although parents’ appraisals may be imagined to exceed those of other evaluators, the existing research controverts this notion. Parents are in fact quite accurate assessors of their children’s giftedness, being more conservative than, and significantly outperforming teachers in, this capacity [30,31,32,33,34]. In one study, for example, 61% of the 118 children felt by parents to be “gifted” (without reference to a specific definition) were found upon subsequent testing to have IQs of 132 or greater (representing the top 2% of the population, a much higher threshold than is typically applied to qualify for a “gifted” designation in school or government settings), and many more were close to this standard [30]; the other studies cited above present accuracy rates consistent with these findings. 

The data presented here derive from a survey of 698 parents and primary caregivers of patients with PANS diagnoses entitled *A retrospective parent-report of onset, interventions, and clinical course of children identified with Pediatric Acute-Onset Neuropsychiatric Syndrome*, the results of which have been published previously [3,5,11,35]. Ninety-two percent of the subjects in this survey resided in the United States; 45 states and the District of Columbia were represented, with the largest fractions of patients deriving from California, New York, and Virginia (7% each).

Among the results previously presented, we demonstrated a significant relationship between *percent days symptom-free* across the disease course (a measure of symptom persistence) and the prevalence of developmental and functional deficits in this population [35]. In the present study, we address the “flip side of the coin” by presenting data on perceived giftedness and aiming to understand factors associated with giftedness. This study is important because gifted children who experience deterioration in cognitive and functional skills may be overlooked and may experience greater delays in diagnosis as they may still perform at a level that is similar to peers. These children may also experience a severe sense of loss if their areas of giftedness are erased during an exacerbation. The following hypotheses were tested:

- Parents/primary caregivers will report higher rates of giftedness in PANS patients than expected based on rates for the general population.
- Rates of perceived giftedness will be negatively associated with PANS symptom persistence.
- Rates of perceived giftedness will be negatively associated with PANS severity.
- Rates of perceived giftedness will be negatively associated with PANS illness duration.

## 2. Materials and Methods

### 2.1. Data Capture

A 146-question, retrospective online survey gathered data from caregivers of children diagnosed with PANS, and from adult PANS subjects, regarding history, symptomatology, interventions, outcomes, and function-related variables. Instrument development is described in Calaprice et al. [3]. The study was approved by the Social and Behavioral Sciences Institutional Review Board at the University at Buffalo. Participants were recruited from PANS patient-advocacy websites, emails, conferences, and *Radio PANDAS Live*, a weekly live internet broadcast related to PANS issue, as well as via posters sent to PANS-treating providers. Respondents electronically certified that a physician had diagnosed PANS or PANDAS, with the following definition offered prior to the certification and electronic consent process: “... *a disorder in which children experience a sudden and severe onset of obsessive-compulsive thoughts and behaviors along with other symptoms that are thought to be precipitated by an infection, environmental trigger, or metabolic disorder. The disorder is described by Swedo and colleagues (2012b)* [1] *and descriptions can be found here.*” (A link then took participants to an NIMH webpage describing PANDAS and PANS that is no longer available.) “*We consider PANS to include PANDAS, which is Pediatric Acute-Onset Neuropsychiatric Disorder Associated with Strep and PITAND, which is Pediatric Infection-Triggered Autoimmune Neuropsychiatric Disorder. Therefore, when we use the term ‘PANS’ we mean PANS, PANDAS and/or PITAND*”.

### 2.2. Data Analysis

A total of 55 of the 753 surveys submitted were excluded from the analysis dataset based on unreliable data, as indicated by failure on one or more predefined logic checks designed to reveal illogical information. To maintain as much consistency in the interpretation of giftedness as possible, the present analysis also included only subjects for whom reporting was done by the primary caregiver, leaving 692 records available for analysis, of which 680 contained answers to one or more giftedness-related questions. Sample sizes varied across questions because some respondents failed to answer some questions and answers of “don’t recall” were excluded from analyses.

The dependent variables in the present analyses are based on answers to the following question: “*Please check any area(s) in which you feel that the patient is particularly gifted*”, which was followed by a list of potential areas of giftedness, a choice of “*Yes, I feel the patient is gifted in this area*” or “*No, I don’t feel the patient is gifted in this area*” and then by a space for comments, labeled *“Please comment on areas in which you feel the patient is gifted, if desired”*. No specific definition for “giftedness” was provided.

The disease-related characteristics studied as independent variables included *length of illness* (calculated from the data as [date of survey response—date of onset of PANS symptoms]), *percent days symptom-free* (a measure of symptom persistence across the full disease course), and *severity of worst episode.* The *percent days symptom-free* variable was derived from answers to the following question: *“During the period from initial onset of PANS symptoms to the present, approximately what percentage of the patient’s days have been symptom-free (treated or untreated)”*? The categorical choices offered were as follows: (a) None, patient has had symptoms every day; (b) 1–10% of days have been symptom-free; (c) 10–25% of days have been symptom-free; (d) 26–50% of days have been symptom-free; (e) 51–75% of days have been symptom-free; (f) 76–99+% of days have been symptom-free; (g) Patient was too young during much of this period to tell; or (h) Don’t remember. Responses b and c were combined into one category, and responses of “patient was too young” and “don’t remember” (g and h) were excluded from these analyses. The *severity of worst episode* variable was derived from answers to the question *“How would you rate the severity of symptoms during the patient’s worst PANS episode (including the initial episode, if that was the most severe)”*?, which was followed by possible responses of (a) None, (b) Minimal (not noticeable to a casual observer), (c) Mild (noticeable to a casual observer, but does not interfere with “normal” performance of age-typical activities), (d) Moderate (interferes with “normal” performance of age-typical activities), (e) Severe (unable to perform age-typical activities), and (f) Incapacitating (interferes with ability to perform basic tasks of daily living). No respondent elected “None” or “Minimal” in response to this question, so only the other choices appear in our Results.

The JMP^®^ statistical program was used to perform quantitative data analysis, with nominal or ordinal logistic regression models used, as appropriate, for categorical variables and ANOVA for continuous variables. There was no significant relationship between age and any type of giftedness, so this variable was not included as a factor in any analyses except those involving duration of illness. Sex bore a statistically significant relationship only to *Creativity*, so it was only included as a factor in analyses related to this dependent variable. Bonferroni corrections for multiple tests were applied as indicated in the table footnotes and text.

Analysis of comments was guided by Braun and Clarke’s [36] approach to thematic analysis. First, two authors (DC and CW) familiarized themselves with the content by reading and re-reading all comments, noting common ideas, and arriving at a consensus list of codes. All comments were then independently coded by each of three authors (DC, CW, RT). Related codes were grouped into themes, which were refined and named, and comments and sample extracts were summarized by the first author (DC). Finally, two authors (CW and RT) reviewed the summary versus the raw data to ensure agreement on counts, as well as that quotations were presented in the appropriate context and that the summary effectively captured the data content.

## 3. Results

### 3.1. Frequency of Giftedness and Associations with Demographic and Disease Traits

**Figure 1:**
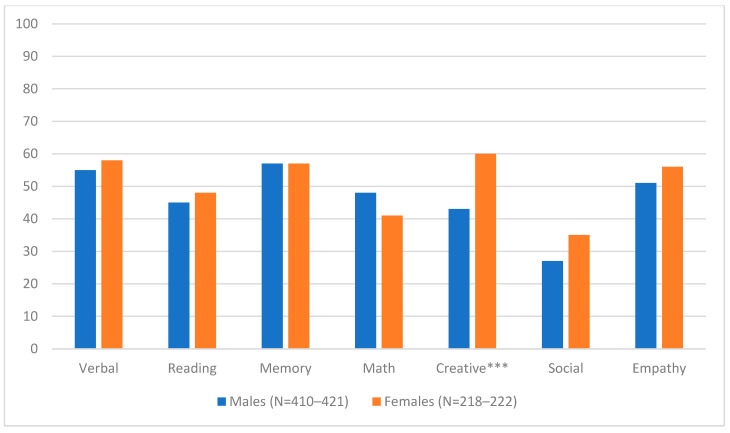
% PANS subjects perceived to be gifted, by sex. *** *p* < 0.001, Bonferroni corrected.

Six-hundred and eighty respondents answered questions about giftedness, the vast majority (>96%) of whom were patients’ mothers (Table 1). Mean patient age was 11.7 years (SD = 4.3, range < 2–35), and mean duration of illness was just over 4 years (Table 1). Rates of reported giftedness ranged from 30% (Social) to 57% (Memory). The proportion of PANS subjects considered to be gifted was fairly evenly distributed across genders, with only creativity, for which 60% of female subjects and <45% of male subjects were perceived as gifted, seeing a significant disparity (Figure 1, Likelihood-Ratio *χ*^2^ = 16.2, DF = 1, uncorrected *p* < 0.0001. Bonferroni-corrected *p* < 0.001). There were no significant relationships between any area of giftedness and any disease metric (illness duration, % days symptom-free, or severity) once Bonferroni corrections were applied. Even without Bonferroni correction, the only significant relationship was between math ability and PANS duration; subjects perceived as mathematically gifted (*N* = 294) had an average PANS duration of 3.9 years, while those not perceived as mathematically gifted (*N* = 347) had an average of 4.4 years (L-R *χ*^2^ = 3.9, DF = 1, *p* < 0.05; Table 1). Notably, this relationship was explained entirely by subjects with ≤75% symptom-free days. Subjects with relatively well-controlled symptoms (i.e., >75% symptom-free days) tended (non-significantly) to have higher rates of mathematical giftedness with increasing length of illness, whereas those with symptoms that were more persistent (≤75% days symptom-free) were significantly less likely to be considered gifted with increasing length of illness (*χ*^2^ = 5.27, DF = 1, *p* = 0.02 without Bonferroni correction).

**Figure 2:**
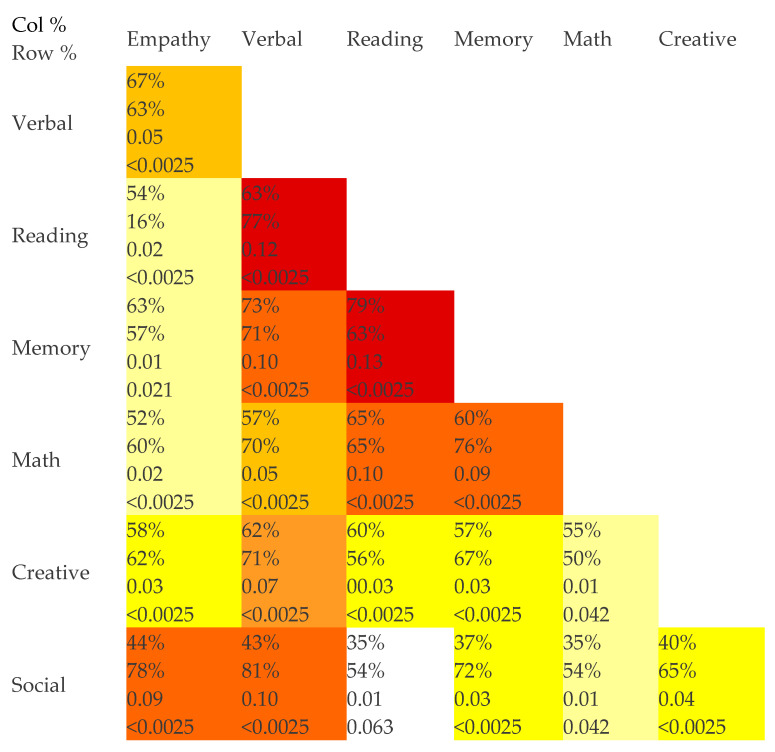
Correlations between areas of giftedness in PANS Subjects. The first number in each cell represents the fraction of subjects in each column category that are also in the respective row category; the second number in each cell represents the reverse. The third number in each cell is the R^2^ for the correlation, and the last number is the Bonferroni-corrected *p*-value for the correlation. Red shading signifies R of 0.12–0.13; dark orange R of 0.09–0.10; dark gold R of 0.06–0.07; light gold R of 0.05–0.06; bright yellow R of 0.03–0.04; light yellow R of 0.01–0.02. White cells represent non-significant correlations once Bonferroni-corrected.

Highly significant correlations were observed between giftedness in almost every pair of areas, with *p* < 0.0025 for all but four pairs (memory/empathy, creative/math, social/reading, and social/math) (Figure 2). The strongest predictors of giftedness in other areas appeared to be verbal giftedness, with R≥ 0.05 between verbal giftedness and all other areas, though memory was also very strongly related to verbal, reading, and math skills.

### 3.2. Themes Among Caregivers’ Comments

Three hundred and forty-one respondents (49%) left comments in the space that allowed for optional elaboration on giftedness. Three themes emerged from these comments: (1) Elaboration on Types of Giftedness; (2) Objective Basis for Perceptions of Giftedness; and (3) Impact of PANS on Giftedness. The paragraphs below summarize themes in the responses, for the sole purpose of giving a flavor for the type of information offered. Because of the entirely open-ended, optional nature of the query, the quantifications offered are highly likely to substantially underestimate the prevalence of the phenomena discussed.

### 3.3. Theme 1: Elaboration on Types of Giftedness

#### 3.3.1. Empathy and Social Skills

In all, 66 of the respondents who stated that their children were gifted in either empathy and/or social skills elaborated with details. Most of these elaborated upon their children’s attunement to the feelings of others, describing them as “attentive to details in social settings”, “gifted in reading emotions”, “highly concerned about other people’s feelings”, and possessing “very high levels of… insightfulness” and an “unbelievable sense of how other people are feeling.” Because of their sensitivity toward others, some subjects were selected by teachers and others to assist or support children with disabilities or other challenges (*N* = 3). Eight respondents claimed that empathy and social awareness declined over time with uncontrolled PANS.

#### 3.3.2. Verbal Abilities and Reading

Elaborations regarding verbal abilities and reading were provided by 106 of the respondents who stated that their children were gifted in one or both of these areas. Some respondents reported their children to have been precocious speakers and readers, with ten reporting reading by age 1–3 and an additional four reporting reading at a very early age, but without providing specifics. Thirteen described exceptionally large and mature vocabularies from very young ages. Reported reading levels were well above grade-level expectation, with eight mentioning reading levels “several” years beyond expectation, four specifically mentioning reading levels 5–11+ years beyond grade level, and more reporting very advanced reading levels without providing specifics.

#### 3.3.3. Memory

Superlatives abounded in respondents’ descriptions of their children’s memories, with 67 respondents elaborating on this area of giftedness. Comments frequently included the words “unbelievable”, “incredible”, “amazing”, “exceptional”, and “remembers everything”. Some children were explicitly described as having “photographic” memories (*N* = 6), while others described skills suggestive of possible photographic memories, for example, the ability to provide detailed driving directions by age 6 or to “remember minute details from events years ago, including what music was playing and what people were wearing.” Respondents uniformly emphasized the extraordinary level of detail in their children’s recall of experiences, books, and facts, even after simply “a glance”. One was able to memorize music with only a few plays; another could recall years of Superbowl teams and scores even though he did not follow football; some had learned multiple languages; and others were able not only to recall past conversations verbatim but to accurately report about details of the surrounding environment. Eleven respondents mentioned that memory—particularly short-term or “working memory”—suffered during a PANS flare; one specifically stated that activities requiring “steps” could be challenging because of difficulty “holding” information short-term.

#### 3.3.4. Creativity

Among the 76 respondents who provided comments in this area, a widespread theme in open-ended responses related to patients’ “out of the box” thinking and “imaginativeness”, terms that were specifically used by seven respondents but traits that were implied or exemplified in most of the other comments. Creativity was reported across multiple endeavors, including but not limited to art, design, music, acting, storytelling/writing, building, and inventing. Four caregivers mentioned that their children’s creative abilities and pursuits persisted during PANS episodes and allowed them to “escape” and/or cope with their PANS challenges, though others mentioned distress at impairment in creative endeavors during exacerbations.

#### 3.3.5. Mathematical Ability

Sixty-three respondents made comments about their children’s giftedness in math, including twelve who provided details about exceptional standardized math test scores and grade levels, with some describing abilities up to seven grade levels beyond expectation. Most of the remainder described exceptional mathematical ability without specific quantification, including four who mentioned children’s abilities to calculate rapidly “in their heads”, in some cases resulting in conflicts with teachers who expected students to “show work”.

#### 3.3.6. Other Abilities

Many respondents provided information about giftedness in areas not addressed by the survey. Most common among these were reports of musical giftedness (*N* = 33), with some noting the ability to play or sing “perfectly” without training and at a young age. Sixteen participants mentioned that their children had the “mind of an engineer”, with gifts in design and construction. Several children were described as “deep thinkers” or “philosophical” (*N* = 4), highly “intuitive” or “sensitive” (*N* = 10), or “funny” and “quick-witted” (*N* = 4). Some were “hyperaware”, with the “amazing ability to see things others don’t”, including, for example, one who “can tell you the speed that the internet is going by looking at the computer screen and can tell you if a pixel is out on a TV”. Several excelled at acting given the ease with which they could memorize lines and insightfully portray their characters.

### 3.4. Theme 2: Objective Basis for Perceptions of Giftedness

Even though this was not requested, 87 respondents used the comments field to provide specific objective evidence of their child’s giftedness. (This number excludes the many others who made references to testing or identification or being “well above grade level” but who did not provide the specific results.) Specific evidence included, for example, test scores, giftedness designations at school, high national or international rankings in activities in which the patients were gifted (e.g., nationally ranked chess champion), and exceptionally high class ranks (e.g., valedictorian). Of these, 16 provided test scores specifically related to intellectual capacity (i.e., IQ). The typical provided IQ score was in the 140–150 range; the lowest was 129. Additional subjects “ran out of questions” or were “off the charts” on tests of intellectual capacity and were deemed to be in a range that exceeded the 99th percentile but that could not easily be quantified.

### 3.5. Theme 3: Impact of PANS on Giftedness

Of the respondents who commented on the impact of PANS on their children’s gifts, eighteen reported that PANS episodes had impacted giftedness only temporarily; thirty-four reported that PANS’ impacts had been apparently permanent (as of the survey date); and four reported that some impacts had been temporary and others permanent. Among those who referenced “permanent” effects, many stated that some level of giftedness eventually returned after PANS-related debility, though not the level present prior to PANS onset. For some, certain abilities remained intact while others deteriorated, but for others, declines were global. Ten parents described children who had previously been gifted and now lagged behind peers in prior areas of strength. Five reported that baseline exceptional strengths were the only reason their children maintained adequate performance in school despite PANS-related deterioration, and two stated that their children’s abilities made it hard to convince school officials that they suffered impairing illnesses.

## 4. Discussion

As has been found for other psychiatric and autoimmune disorders, PANS appears in our study to be associated with significantly higher rates of caregiver-reported giftedness than expected based on population prevalence, suggesting that the phenomenon of giftedness in PANS deserves further, more systematic exploration. Nationwide, the fraction of public-school children designated “Gifted or Talented” during the time of our survey was approximately 6.7% [37]. Even if we were to “correct” our study’s giftedness estimates of 30–57% by a factor representing the most extreme published rates of parents’ overestimation of giftedness (approximately 50%), this would yield “adjusted estimates” in our sample of approximately 20–40% *per specific area of giftedness*, still substantially exceeding not only 6.7% *(for all areas of giftedness combined),* but even the most liberal estimated population rates. Further support for the likely veracity of our findings comes from the detailed comments provided by parents citing objective evidence of their children’s giftedness. Although we took no measures to verify the parents’ assertions and there is a risk of parents misreporting details, the risk is minimized by this being an anonymous survey with no potential benefit to, or even direct contact with, the survey respondents or their children. Further, since the comments were offered spontaneously by respondents rather than solicited systematically, the quantification of themes we provided is highly likely to underestimate the prevalence of the mentioned phenomena.

The specific patterns of giftedness revealed in our data suggest that exceptional memory may be a core element of the giftedness that characterizes PANS children. This finding stands in contrast with the pattern seen in the population at large, whereby intellectually gifted children generally do not possess superior memories to children of typical intellect (reviewed in [38]). Not only was memory the most commonly reported area of giftedness in our sample and the most emphatically described, but it was also highly likely to be associated with other types of giftedness. This is perhaps not surprising. Descriptions of verbal intelligence in our sample generally involved use of an expansive vocabulary, together with the ability to fluently summon apt language—skills based on the ability to store and retrieve information. Mathematical ability, particularly at the level of math taught to children (e.g., “math facts”, times tables, basic computation), is also partly a function of the ability to proficiently store and retrieve information. That memory and mathematical ability were the areas most often reported to deteriorate with PANS disease activity further suggests the possibility that memory-related differences may characterize the brain activity of PANS children.

Social giftedness in our study was less commonly reported than other talents, though still present at a remarkably high rate given that previous studies have predominantly highlighted the social challenges experienced by PANS patients [6,10]. In our frequency data and in the comments, social giftedness in our subjects was often linked to exceptional abilities to perceive the feelings of others, to empathize, and to self-express. To what extent empathy in PANS children results from an innate sensitivity to the feelings of others versus the experience of challenging and painful PANS episodes that foster the ability to relate to the experiences and emotions of other people is unknown. In either case, this “flip side of the coin” is important to our understanding of PANS patients. It is possible that the previously reported social challenges represent not neurological disturbances but rather the practical impacts of PANS disease activity on social opportunities and development; it is also possible that intellectual giftedness may be a previously unappreciated factor in perceived social disinterest or discomfort. Even in the absence of PANS, extraordinary abilities often set children apart from their peers in many ways, including introversion, perceptions of being “different”, and an inability to find peers sufficiently stimulating; this is particularly true for gifted children with creative tendencies, as was often reported for our PANS subjects (e.g., [38,39,40,41]).

A prominent theory to explain the associations between giftedness and psychiatric and autoimmune diagnoses is that highly intelligent individuals possess unique psychological and physiological “overexcitabilities” that can be both enabling and disabling, either at the same time but in different domains, or alternately based on emotional state, physiological state, or level of stimulation [13,41,42,43,44,45,46,47,48,49]. The term “overexcitability” (translated from the Polish ‘nadpobudliwosc’) was first introduced by Kazimierz Dabrowski, a Polish psychiatrist who studied individuals with high cognitive ability across their lifespans [49,50,51,52,53]. Compared to individuals with average or lower IQ, Dabrowski found the intellectually gifted to have uniquely “hyper-reactive”, intense ways of experiencing and responding to their environments across multiple domains, including sensory, psychomotor, imagination, intellect, and emotions. In addition to shaping personalities and abilities generally, the broad neurological overexcitability experienced by gifted individuals can be associated with tendencies toward depression, anxiety disorders, and tics, as well as with “overexcitability” of the closely intertwined immune system, resulting in exaggerated immune reactions to stimuli [14,54,55]. That our PANS subjects frequently demonstrated giftedness across multiple domains, along with the classic PANS traits of sensory hypersensitivity, psychomotor symptoms, emotional dysregulation, and an overactive immune response [3], is interesting in this context.

Also of interest in our sample was that giftedness as perceived by parents was generally robust to the PANS disease experience. Within the sample as a whole, we found no statistically significant relationships between any category of giftedness and either disease severity or symptom persistence. Among the subset of subjects with poorly controlled disease (as reflected in moderate to high levels of symptom persistence), the rate of perceived mathematical giftedness declined with increasing disease duration, though this relationship was not statistically significant once Bonferroni corrections were applied. It is possible that giftedness in one or more areas generally persists even in the context of PANS disease activity; many of the parents’ comments supported this, though others suggested the opposite. Also possible is that parents’ perceptions of giftedness, which may have developed over their children’s entire lives, are not inclined to change even in the face of PANS-related deficits, which may be perceived as aberrations and not core to who their children are (i.e., “state” rather than “trait”). Unlike most other facets of giftedness addressed in this study, math is largely taught through schooling rather than absorbed naturalistically, and giftedness is largely measured by objective exams. For this reason, loss of mathematical giftedness over the disease course could represent simply a cumulative “falling behind” due to disease-related nonattendance (or inattention) at school among subjects with relatively high symptom persistence, and loss of math ability could be more evident to parents—and harder to deny—than loss of other abilities. Alternatively, particularly given that loss of math ability is a hallmark symptom of PANS episodes, PANS disease activity may have specific negative impacts on the brain areas or functions involved in math, and loss of giftedness over time may represent damage to these areas over the course of poorly controlled illness.

Our findings have several clinical implications. From a psychotherapeutic point of view, it may be important to recognize that the heightened sensitivity, perfectionism, and need for control often seen in gifted children may create particularly high levels of distress for those experiencing PANS, as they struggle to deal with uncontrollable and often embarrassing symptoms that impede their ability to perform as they expect. Although the severity of symptoms varies among children with PANS, the cognitive challenges, including memory, concentration, executive function, processing speed, and speech difficulties, are likely to be particularly unnerving for gifted children who are used to relying on these as areas of strength. Gifted children and adolescents may also be particularly likely to extrapolate from their current challenges to implications for their future ambitions, causing another layer of distress that could be addressed in the therapeutic process. Psychotherapies such as cognitive behavioral therapy are generally recommended for all PANS patients [56] but may be particularly critical for those who are gifted, even when school and social performance appear adequate.

Giftedness may also impact the process of diagnosis and ongoing assessment. For example, children may experience quirks or eccentric behaviors that are initially attributed to their intelligence, potentially delaying a correct diagnosis of PANS. Gifted children may also be unusually perceptive about the impacts of their condition on their families and on their own opportunities (for example, opportunities to go to camp or attend college away from home), as well as skilled at masking their symptoms, making it difficult for parents and professionals to accurately ascertain the severity of their condition. Having a gifted child perform at an age-typical level during exacerbation also increases the likelihood of misdiagnosis and underdiagnosis of the condition. Delayed diagnosis and treatment in PANS has been associated with more persistent symptoms [3].

As a community-based survey, this study’s limitations include self-selection of participants and self-reporting of diagnoses, disease-related traits, and giftedness. Aware of these possible sources of bias, we endeavored to capture a sample that broadly represented the population of PANS patients in the US, recruiting not only at specialist practices but also utilizing the mailing list of PANDAS Network (the most visited website for PANDAS/PANS searches) and publicizing the study through a weekly live internet broadcast related to PANS issues. The survey was described as relating to PANS traits generally, and since the topic of giftedness received no mention during recruitment, we have no reason to believe that we preferentially captured parents to whom giftedness was of particular interest. Although we did not request medical records to confirm the diagnosis or disease-related traits, PANS/PANDAS diagnostic criteria were provided during the Informed Consent process and subjects were required to actively confirm that the diagnosis had been made by a physician. Respondents reported anonymously and received no payment or other incentive to complete the time-consuming survey, presumably further minimizing participation by subjects not meeting diagnostic criteria. Symptoms associated with PANS disease activity were detailed throughout the survey; specific guidelines were provided for severity level choices; and we allowed “don’t know” or “don’t recall” responses to questions to relieve any pressure to guess about poorly recalled variables. As noted earlier, subjects who did not feel confident that they could answer questions accurately, or who were revealed through logic checks to be poor reporters, were excluded from the analyses.

One possible source of confounding is related to the fact that PANS is a rare, complex, historically controversial, and presumably underrecognized condition. Given this, it is likely that parents who find their way to a diagnosis for their children are not characteristic of the population whose children actually suffer from PANS, but rather represent only the portion with access to specialist providers (including both the insurance and/or financial resources to see them, and the ability to travel to them) and/or with the intelligence, education, drive, and communication skills to perform their own research and often convince skeptical non-specialists to consider the diagnosis. It is likely that parents with these traits will more commonly have gifted children than parents in the population at large. This possibility notwithstanding, our sample is likely quite representative of the population of PANS patients who are in fact diagnosed, and our results should thus pertain to the population of patients being seen in medical practices and in schools. Although we did not obtain data relating to socioeconomic status or education level in our survey, the clinical characteristics of our PANS sample align with the characteristics of other study samples (e.g., gender and age distribution, time to diagnosis, range of acuity of onset, frequency of presenting symptoms, recurrence rates; [2,57,58,59]), further supporting the relevance of our findings to the clinical population.

## 5. Conclusions

Recognition of giftedness in PANS patients is important for multiple reasons. First, in terms of patient management, it is only by recognizing potential giftedness that we can adequately identify disease-related impairments that may persist despite the ability to function adequately in school and other settings. A gifted child may operate at or above grade level even when significantly impaired, leading teachers, doctors, therapists, and others not to recognize impairment and hence not to appreciate the inadequacy of treatment or the need for accommodations. This may be compounded by parents’ discomfort discussing how their currently impaired children “are actually gifted”, and/or insisting that impairment persists even when performance exceeds standards. To counteract this dynamic, parents should be encouraged to openly discuss their children’s pre-PANS abilities and should not be assumed to exaggerate.

On the flipside, caution must also be exercised to not over-medicalize this population. Gifted children frequently exhibit some of the same traits that may characterize PANS, such as perfectionism, boredom and inattentiveness in school, high activity levels, disinterest in “typical” childhood play, sensory hypersensitivity, and “obsessive” levels of interest in specific subjects. It is important to recognize that such traits may be inherent aspects of a gifted child’s personality rather than representing disease activity, again underscoring the need for a thorough and open discussion of pre-PANS function, behavior, milestone achievement, etc., so that a child is not made to feel “abnormal” where no abnormality (beyond the giftedness itself) exists. Indeed, such traits may promote the success of the gifted child and can in some cases be celebrated as “superpowers” rather than lamented as dysfunction, increasing child and parent morale and shaping the approach to supportive therapy. Finally, it is only by recognizing the baseline potential of this population that we can appreciate the true cost of failures to diagnose and effectively treat this often misunderstood, under-recognized, and inadequately treated condition.

## Figures and Tables

**Table 1:** Characteristics of PANS subjects perceived and not perceived by primary caregivers to be gifted, by gifted category (*N* = 680).

	Empathy		Verbal		Reading		Memory		Math		Creativity		Social	
Category/ Trait	Gifted	Not Gifted	Gifted	Not Gifted	Gifted	Not Gifted	Gifted	Not Gifted	Gifted	Not Gifted	Gifted	Not Gifted	Gifted	Not Gifted
**Total *N* (%)**	336 (53)	301 (47)	360 (56)	280 (44)	294 (46)	344 (54)	359 (57)	273 (43)	294 46)	347 (54)	312 (48)	321 (52)	188 (30)	445 (70)
**% Reporters = Mother**	96	96	96	96	96	95	95	97	96	95	96	96	96	97
**Age** **Mean (SD)**	11.8 (0.24)	11.5 (0.25)	11.6 (0.23)	11.6 (0.26)	11.6 (0.25)	11.6 (0.23)	11.4 (0.23)	11.8 (0.26)	11.4 (0.25)	11.8 (0.23)	11.9 (0.25)	11.4 (0.24)	11.3 (0.32)	11.7 (0.20)
**PANS duration, mean (SD)**	4.2 (0.21)	4.2 (0.22)	4.3 (0.20)	4.0 (0.23)	4.3 (0.22)	4.1 (0.21)	4.0 (0.20)	4.3 (0.23)	3.9 * (0.22)	4.4 (0.21)	4.2 (0.22)	4.2 (0.21)	3.8 (0.28)	4.2 (0.18)
**Days symptom- free, *N* (%)**														
*None*	63 (20)	52 (19)	66 (19)	48 (19)	55 (20)	58 (18)	70 (21)	42 (17)	56 (20)	57 (18)	60 (21)	51 (17)	32 (17)	82 (20)
*1–25%*	82 (26)	86 (31)	96 (2)	74 (29)	78 (28)	89 (28)	91 (27)	78 (31)	74 (26)	94 (29)	82 (28)	86 (28)	42 (23)	128 (31)
*26–50%*	55 (17)	45 (16)	54 (16)	46 (18)	43 (16)	56 (18)	57 (17)	41 (16)	48 (17)	53 (17)	52 (18)	47 (16)	39 (21)	60 (15)
*51–75%*	58 (18)	46 (17)	56 (17)	49 (19)	42 (15)	63 (20)	64 (19)	40 (16)	42 (15)	61 (19)	52 (18)	54 (18)	32 (17)	72 (18)
*76%+*	63 (20)	48 (17)	67 (20)	42 (16)	59 (21)	54 (17)	59 (17)	50 (20)	62 (22)	54 (17)	43 (15)	64 (21)	39 (21)	68 (17)
**Most severe episode *N* (%)**														
*Mild*	16 (6)	9 (3)	14 (4)	9 (4)	10 (4)	14 (5)	12 (4)	13 (5)	9 (4)	15 (5)	10 (4)	14 (5)	7 (4)	17 (4)
*Moderate*	50 (17)	59 (22)	64 (20)	50 (20)	55 (22)	57 (18)	58 (19)	52 (21)	46 (18)	65 (21)	49 (18)	61 (21)	33 (20)	75 (19)
*Severe*	86 (30)	83 (31)	94 (29)	79 (32)	71 (28)	100 (32)	95 (30)	76 (31)	83 (33)	90 (29)	79 (29)	93 (33)	49 (30)	122 (31)
*Incapacita-ting*	139 (48)	119 (44)	148 (46)	108 (44)	116 (46)	138 (45)	148 (47)	103 (42)	115 (45)	141 (45)	137 (50)	118 (41)	73 (45)	182 (46)

* *p* ≤ 0.05.

## Data Availability

Deidentified data and question text that directly pertain to the analyses included in this manuscript are available upon request.
